# Boron nitride-enabled printing of a highly sensitive and flexible iontronic pressure sensing system for spatial mapping

**DOI:** 10.1038/s41378-023-00543-x

**Published:** 2023-05-26

**Authors:** Zekun Yang, Qikai Duan, Junbin Zang, Yunlong Zhao, Weihao Zheng, Ran Xiao, Zhidong Zhang, Liangwei Hu, Guirong Wu, Xueli Nan, Zengxing Zhang, Chenyang Xue, Libo Gao

**Affiliations:** 1 Key Laboratory of Instrumentation Science and Dynamic Measurement Ministry of Education, North University of China, 030051 Taiyuan, China; 2 School of Automation and Software Engineering, Shanxi University, 030006 Taiyuan, China; 3 Department of Mechanical and Electrical Engineering, Xiamen University, 361102 Xiamen, China; 4 School of Mechano-Electronic Engineering, Xidian University, 710071 Xi’an, China; 5 Department of Mechanical Engineering, City University of Hong Kong, 999077 Kowloon, Hong Kong SAR

**Keywords:** Sensors, Electrical and electronic engineering

## Abstract

Recently, flexible iontronic pressure sensors (FIPSs) with higher sensitivities and wider sensing ranges than conventional capacitive sensors have been widely investigated. Due to the difficulty of fabricating the nanostructures that are commonly used on electrodes and ionic layers by screen printing techniques, strategies for fabricating such devices using these techniques to drive their mass production have rarely been reported. Herein, for the first time, we employed a 2-dimensional (2D) hexagonal boron nitride (*h*-BN) as both an additive and an ionic liquid reservoir in an ionic film, making the sensor printable and significantly improving its sensitivity and sensing range through screen printing. The engineered sensor exhibited high sensitivity (S_min_> 261.4 kPa^−1^) and a broad sensing range (0.05–450 kPa), and it was capable of stable operation at a high pressure (400 kPa) for more than 5000 cycles. In addition, the integrated sensor array system allowed accurate monitoring of wrist pressure and showed great potential for health care systems. We believe that using *h*-BN as an additive in an ionic material for screen-printed FIPS could greatly inspire research on 2D materials for similar systems and other types of sensors.

**Figure:**
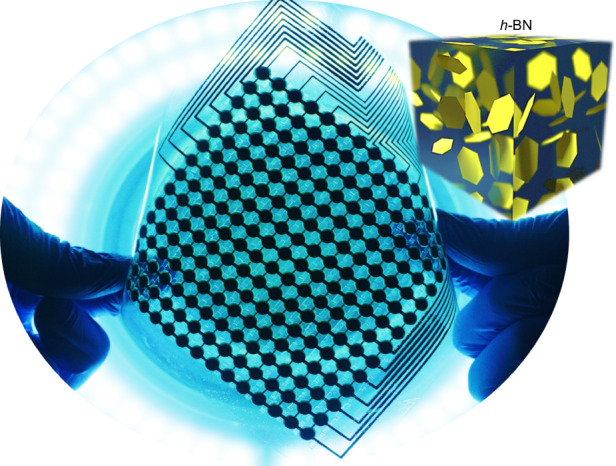
Hexagonal boron nitride (*h*-BN) was employed for the first time to make iontronic pressure sensor arrays with high sensitivity and a broad sensing range by screen printing.

Hexagonal boron nitride (*h*-BN) was employed for the first time to make iontronic pressure sensor arrays with high sensitivity and a broad sensing range by screen printing.

## Introduction

Flexible pressure sensors are widely applicable in health care monitoring^1–5^, robotic electronic skin^6–9^, and wearable electronics^10–13^ due to their attributes of high mechanical conformability and softness to the human body. Specifically, flexible iontronic pressure sensors (FIPSs) have attracted much attention. FIPSs have inherently high specific capacitance and superior sensitivity due to the super capacitive effect at the electrode/ionic film interface, differing from conventional plane parallel capacitance^14–20^. Therefore, FIPSs address the typical issue of parasitic noise caused by physical motions that are prevalent in capacitive sensors. However, the lack of methods for producing FIPS arrays with high sensitivities and wide sensing ranges on a large scale suppresses their practical application to the real world from the laboratory.

Similar to conventional plane parallel capacitive sensors, the construction of microstructures on the ionic layer interface can significantly improve the sensitivity of the sensor over a wide range^21–24^. Typically, the construction of micropatterns, porous structures, and multilayers, in addition to combinatorial strategies, improves the sensitivity, sensing range, low limit of detection (LOD) and response time characteristics of the sensors. Almost all of these approaches are based on the principle that the sensor is initially in a mechanically unstable state and susceptible to deformation when subjected to mechanical pressure. The micro/nanostructure in the initial state can form a mechanically unstable configuration at the interface between the ionic layer and the electrode layer, which is extremely unstable when subjected to pressure loading; this instability results in sudden increases in contact area and capacitance and a stable linear capacitive output, even in the saturated state. For instance, Guo et al. and our group previously used abrasive paper as a mold to build a structured polyvinyl alcohol (PVA)/H_3_PO_4_ ionic film to make an FIPS with a sensitivity of 220 kPa^−1^ and a detection range of 0.08 Pa–360 kPa, revealing the advantages of the internal filling structure^25–27^. Liu et al. demonstrated that a polyurethane (PU)–ionic liquid (IL) composite foam layer with high porosity (95.4%) not only has a low Young’s modulus and good compressibility but also produces an FIPS with high sensitivity (9280 kPa^−1^)^28^. Specifically, Xiao et al. was the first to use multilayer structures in the sensor to achieve a sensitivity of 9.17 kPa^−1^ over 2 MPa^2^. Despite such high performance in terms of the sensitivity and sensing range achieved, the microfabrication of ionic films is always tedious (involving steps such as molding) for large-scale fabrication and practical application. Additionally, screen printing is a proven method for fabricating large-scale flexible electronic devices at the industrial level^29,30^. However, less research has been reported on the use of screen printing to fabricate electrodes and microstructured ionic films for FIPS. Therefore, in-depth research is needed to facilitate practical applications of iontronic sensors.

Herein, we present for the first time a screen printing strategy for preparing FIPS arrays by introducing hexagonal boron nitride (*h*-BN). We employ *h*-BN as an additive to ionic ink, thus enabling the printing of microstructured ionic films and electrodes on flexible substrates. Due to this simple yet effective method, we achieve FIPSs with excellent sensitivities (*S*_min_> 261.4 kPa^−1^), wide sensing ranges (50 Pa–450 kPa) and long-term cycling capabilities (more than 5000 cycles without signal degradation at a high pressure of 400 kPa). In addition, the fabricated flexible pressure sensor array can be used as a smart mouse pad to help people accurately monitor wrist pressure during long working hours. This work demonstrates the practical potential of our integrated sensor device using screen printing.

## Results and discussion

### Device fabrication and structural characterization

**Fig. 1:**
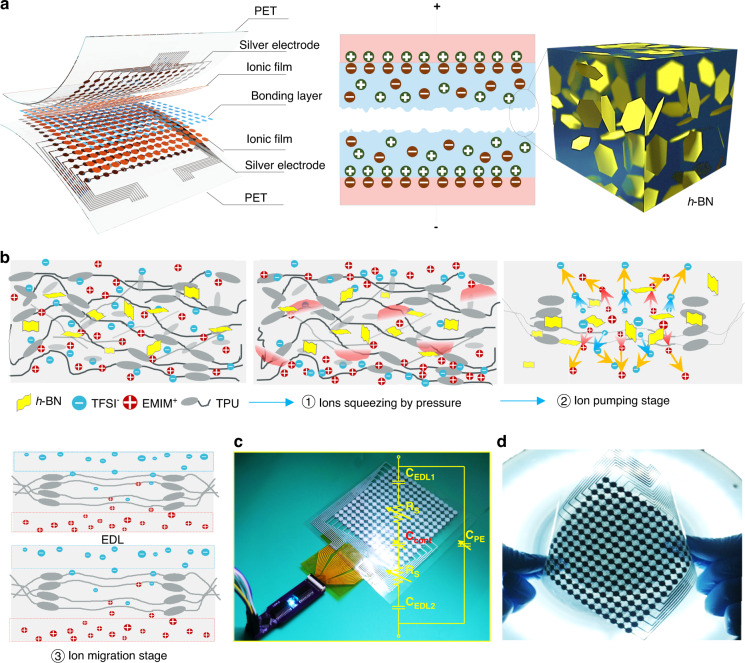
Device design and implementation. **a** Schematic illustration of the FIPS. **b** Sensing mechanism of the FIPS. **c** Digital optical image of the sensor arrays; the inset is the corresponding equivalent circuit. **d** Sensor that is easily mechanically bent

Spatial mapping through flexible sensing arrays is an effective approach for health care monitoring and gesture recognition. However, manufacturing such flexible and wearable pads is difficult and expensive via conventional microelectromechanical system (MEMS) approaches. In particular, due to interferences from electrostatic charges from the human body, it is often difficult for such pads to accurately generate signals when mounted on skin. In this study, we propose the realization of a practical application of a flexible sensor for carpal tunnel syndrome for a typical mouse used by people. The sensing system is capable of 2D and 3-dimensional (3D) wrist pressure spatial mapping through an integrated pressure sensor system that consists of an iontronic pressure sensor array, a circuit board, and host computer software (Fig. S1). Figure 1a shows a schematic illustration of the composition of the sensor device. Silver is selected as the electrode and is screen printed on a flexible polyethylene terephthalate (PET) substrate, while thermoplastic polyether urethane (TPU)–ionic liquid (ILD)-*h*-BN serves as the ionic film. The sandwich structure is then combined by a bonding layer and a hot-pressing process to form the iontronic pressure sensor device. The novelties of this device are as follows. First, a screen printing method is used to fabricate the sensor for practical applications. In this process, both the electrode and the ionic layer can be printed directly on the substrate, which greatly reduces the manufacturing difficulty and cost. More importantly, micropatterned structures on the ionic film can be formed during the printing process instead of using the conventional templating method. Second, the doping of *h*-BN can not only serve as a doping material to make the ionic layer printable but also improve the sensor performance through the ion pumping effect and the electrolyte reservoir, as shown in Fig. 1b. Specifically, in the initial state, *h*-BN, the cations and anions of the ionic liquid, and TPU are homogeneously mixed. Some of the ions are adsorbed onto the surface of *h*-BN due to hydrogen bonding effects, while the high specific surface area of *h*-BN allows for more ion adsorption. Additionally, when the ionic film is subjected to mechanical deformation, the weak hydrogen bonds between the ions and *h*-BN are broken, and the ions are ejected^31,32^. On the one hand, *h*-BN can effectively reduce the viscoelasticity of sensitive materials and increase the stiffness of TPU, thus reducing the hysteresis effect. On the other hand, *h*-BN can reduce the crystallinity of the polymer, form ion transport channels and shorten the ion conduction path, thus improving the sensitivity of the sensor. This process is known as the ion pumping phase, which subsequently generates high electrical double-layer (EDL) capacitance. Benefiting from this rational design, the sensor shows excellent sensitivity and sensing range performance. The inset in Fig. 1c shows the corresponding equivalent circuit, and the main capacitance is derived from the contact capacitance (*C*_cont_). Figure 1c shows a digital optical image of a screen-printed iontronic pressure sensor array. The sensor array has 15 × 15 sensing units connected to the circuit board by laser-cut copper electrodes with a total size of 11 × 11 cm^2^. In addition, the flexible sensor array is easily bent and mechanically stable, which proves its versatility for applications (Fig. 1d).

**Fig. 2:**
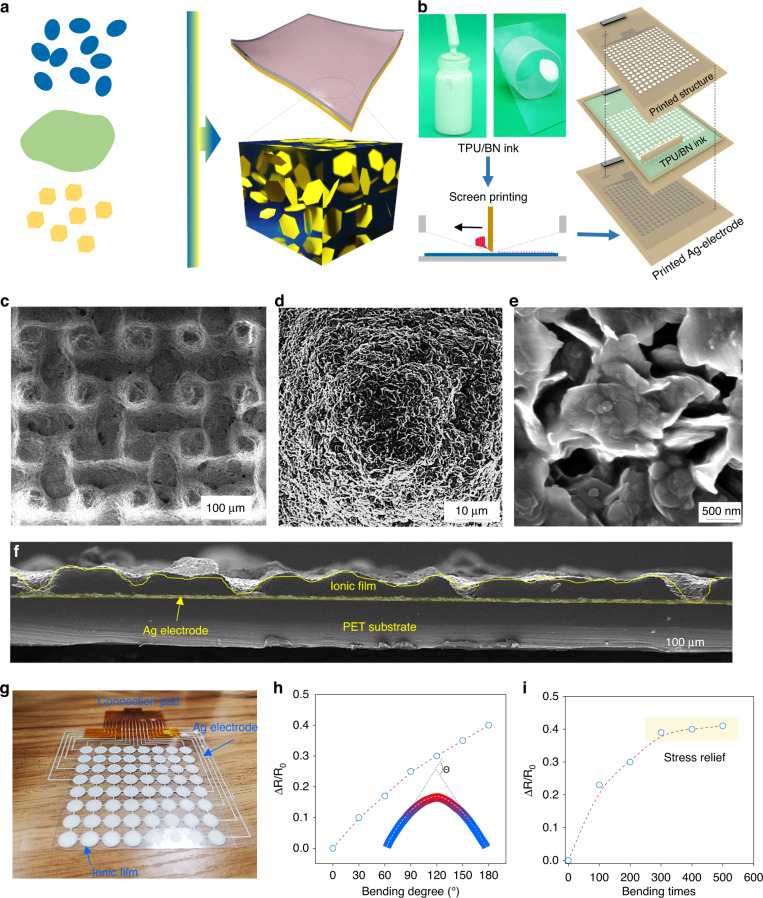
Fabrication and structural characterization of the sensor. **a** Composition of TPU-based ionic ink. **b** Fabrication of the silver electrode and ionic film by screen printing. **c**–**e** Top view and **f** cross-sectional view of the ionic film. **g** Digital optical image of the electrode and ionic film on PET. **h** Resistance variation in the electrode and ionic film bent to various degrees. **i** Resistance variation in the electrode and ionic film bent for 500 cycles

Screen printing is a cost-effective and additive method for fabricating large-scale flexible electronic devices. The film manufactured by screen printing is made by stacking many squares of material through very small mesh holes. The thickness of the film can be tuned by the amount of screen stacking in a similar manner to 3D printing, which requires a high-viscosity printing material. The introduction of *h*-BN nanosheets as a viscosity thickener allows the ink to meet the requirements of screen printing. *h*-BN is the most common BN-stabilized phase among the four phases of *r*-BN, *c*-BN, and *w*-BN, where N and B atoms are connected by σ covalent bonds between the same layer and by van der Waals forces between different layers^33^. TPU fragments, ionic liquid and *h*-BN nanosheets are mixed by magnetic stirring in *N*,*N*-dimethylformamide (DMF) (Fig. 2a, S2, and S12). *h*-BN doping is essential for controlling the printing quality of flexible sensors. A low doping mass results in excessive slurry flow and a printed film thickness that is overly thin and cannot form the desired structure. Additionally, an overly high doping mass can lead to poor film formation and voids. Both cases can lead to short circuits at the contacts of the upper and lower electrodes of the sensor when under pressure. Figure S3 and Fig. 2b show the ink states when 1.0 g, 1.5 g and 2.0 g are added to the TPU solvent. When the same amount of ink is added dropwise to a glass plate tilted at 45°, 1 g and 1.5 g *h*-BN of slurry easily slide off the substrate, which is not in accordance with the printing requirements. In contrast, 2 g of *h*-BN powder doping can meet the optimal printing conditions without significant slipping. Therefore, 2 g of *h*-BN is used to prepare the ionic film. In addition, the conductive silver paste is screen-printed directly onto the plasma-cleaned PET substrate as the electrode. The ionic layer is then printed and cured after heating at 80 °C for 30 min. Detailed information can be found in Fig. 2b, S4, and S15. The microstructure of the ionic film is investigated by scanning electron microscopy (SEM), as shown in Fig. 2c–e. Interestingly, we observe that the micropillars are successfully fabricated, and the surfaces of the micropillars are rough, which is favorable for improving the sensitivity of the sensor. The microstructure is mainly formed when the printed paste passes through the small holes of the screen. This ionic layer with a unique microstructure is formed because the paste is sufficiently viscous to not flow easily. The cross-sectional view further confirms the microstructure and sandwich-like configuration shown in Fig. 2f. The unique structure of the ionic film that can completely cover the silver electrode pattern is further demonstrated. The microstructure forms due to the optimal *h*-BN doping and the suitable mesh size, which is discussed later. Digital optical images of the prepared electrode pattern and ion film are shown in Fig. 2g. The radius of the electrode pattern of the sensor unit is 5 mm, and the width of the silver electrode lead is 500 µm. Notably, the ionic film must completely cover the electrode pattern (Fig. S5), and the reason for printing the ionic film only on the electrodes rather than directly on the whole PET substrate is that such a design can greatly reduce the crosstalk caused by mechanical coupling. In addition, we test the electrical properties of the printed electrodes with ionic layers under bending. The resistance of the electrode increases with increasing bending angle due to an increase in the tensile strains of the silver electrode and the ionic film with increasing bending angle (Fig. 2h). Furthermore, the electrical resistance values of the electrode when bent 180 degrees for different numbers of bending cycles are determined. Although the resistance increases at the initial stage as the number of bending cycles increases from 100 to 300, it stabilizes after 300 to 500 cycles. This stabilization may be due to the release of the internal stress of the silver electrode and the ionic film as the number of bends increases (Figs. 2i and S6).

### Sensor characterization

**Fig. 3:**
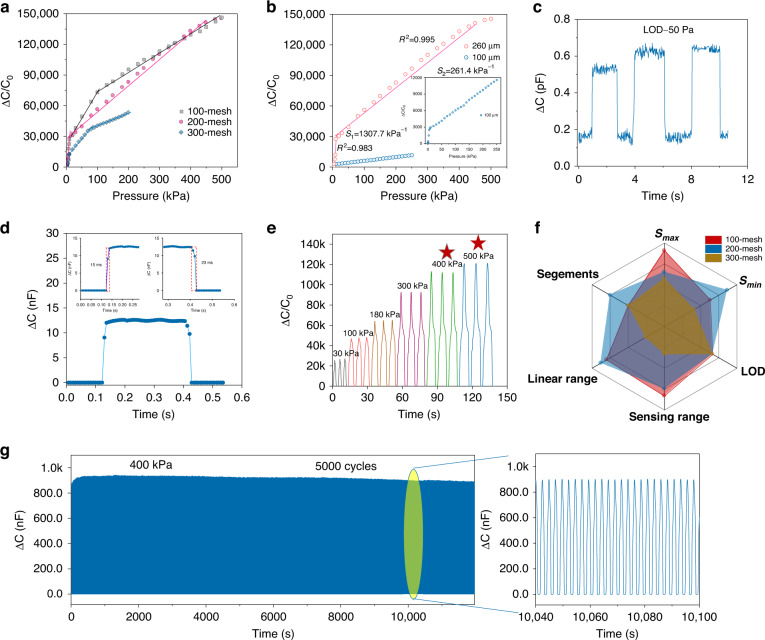
Sensor characterization. **a** Capacitance variation in sensors prepared by different sized meshes. **b** Capacitance variation in sensors prepared by different bonding layers. **c** LOD of the sensor. **d** Response time of the sensor. **e** Capacitance variation in sensors at a continuous pressure. **f** Performance comparison of the sensor prepared by different sized meshes. **g** Long-term cycling ability of the sensor at 400 kPa for 5000 cycles

As shown in Figs. 3 and S7, the performance characteristics of the sensor, such as sensitivity, sensing range and linearity, determine its applicability. The sensitivity is determined by *S* = (∆*C/C*_0_)*/*∆*P*, where *S* is the sensitivity, ∆*C* is the capacitance variation, ∆*P* is the pressure change, and *C*_0_ is the initial capacitance value of the sensor in nonpressure mode. The initial capacitance of the sensor fluctuates at approximately 4.0 pF due to the existence of a bonding layer. The screen mesh pore size is very important in the screen printing preparation process, which determines the sensitivity and printable quality of the sensor. Herein, various mesh sizes of 100, 200 and 300 are investigated. Figure 3a shows the variation in capacitance as a function of pressure. The capacitance of the printed sensor changes rapidly to a pressure of 10 kPa, mainly due to the bonding layer used in this study; these results are similar to those of our previous study^27^ (Fig. S8). For the 100-mesh printed sensor, the linear interval is divided into three periods, with a maximum sensitivity (*S*_max_) of 1822 kPa^−1^ and minimum sensitivity (*S*_min_) of 190 kPa^−1^. For the 200-mesh printed sensor, the *S*_max_ is 1307.7 kPa^−1^ below 10 kPa and 261.4 kPa^−1^ from 10 kPa to 450 kPa. However, for the 300-mesh sensor, although the sensor has three segments, the sensing range is severely limited to 200 kPa, and *S*_max_ and *S*_min_ are 1163 and 124 kPa^−1^, respectively. Clearly, the sensing range decreases as the mesh number increases from 100 to 300. This decrease occurs because for the screen printing process, the larger the mesh size is, the smaller the mesh hole. This small size results in less material passing through the mesh hole, decreasing the thickness of the printed ionic film. Furthermore, the FIPS prepared from the 200-mesh screen clearly shows high linearity, high sensitivity and a wide sensing range. Furthermore, for the flexible sensor package, the sensor configuration is shown in Fig. S8. Notably, the sensors are bounded together by 3 M double-sided adhesive tape (Minnesota Mining and Manufacturing Company). The thickness of the tape plays an important role in improving the overall performance levels of the sensors, which is usually ignored by other studies. The thickness of the double-sided adhesive tape is controlled at 260 µm and 100 µm for the sensors made by 200-mesh screen printing. Clearly, the 260-µm bonded sensor shows a wider sensing range and higher sensitivity than the 100-µm sensor (Fig. 3b). The device shows high linearities of 0.983 and 0.995 in the sensing range. Figure S8 clearly shows that when 260-µm or 100-µm double-sided tape are used as encapsulation layers, the air spacer layer thicknesses are approximately 200 or 40 µm, respectively. Therefore, the initial capacitance of the sensor with a 40-µm spacer layer thickness of is larger than that of the sensor with a 200-µm spacer layer thickness; therefore, the sensitivity is significantly reduced. Moreover, the low thickness limits the compressive space, reducing the sensing range. Therefore, we use a 260-µm bonding layer as the adhesive film for the sensors, considering the sensitivity and linear sensing range. In addition, the sensor shown in Fig. 3c has a low limit of detection of 50 Pa, demonstrating its excellent sensitivity to pressure sensing. To determine the LOD of the sensor, a series of pressure samples are prepared using known standard pressure devices, including pressures lower than the expected minimum LOD of the sensor. During the experiments, each standard sample is applied to the sensor, and the output signal, such as the voltage or current, of the sensor is recorded. The recorded output signals are statistically analyzed to calculate the average output signal and standard deviation for each pressure. Using the data obtained from the statistical analysis, the LOD of the sensor can be calculated. Typically, the LOD is defined as the minimum pressure value that can be statistically distinguished from zero. As shown in Fig. 3c, we characterize the low detection limit of the sensor multiple times. Furthermore, Fig. S16 demonstrates that at pressures of 20 Pa and 40 Pa, the response of the sensor is difficult to distinguish due to noise interference. However, at a pressure of 50 Pa, the response generated by the sensor is approximately 0.57 pF, with a significantly improved signal-to-noise ratio, providing further validation that the low detection limit of the sensor is approximately 50 Pa. Figure 3d shows that the screen-printed sensor has a fast response time of 15 ms and a recovery time of 23 ms at a continuous pressure of 5 kPa, demonstrating an extremely fast response. Moreover, by applying pressures of 30 kPa, 100 kPa, 180 kPa, 300 kPa, 400 kPa, and 500 kPa to the sensor, the sensor can output a stable capacitive signal (Fig. 3e). The data further demonstrate that the sensor remains stable and outputs a relatively constant capacitance signal even under high pressures of 400 kPa and 500 kPa. We compare the various parameters of our prepared sensor, as shown in Fig. 3f. The sensor prepared by the 200-mesh screen performs relatively well in terms of sensitivity, sensing range, LOD and linear sensing ranges and segments, determining its practical capability and the simplicity of its circuit processing; therefore, the sensor prepared by 200-mesh screen printing is mainly used in this study. Long-term stability is imperative for practical applications, and the sensor shows no significant degradation even after 5000 pressure cycles at 400 kPa and after 8000 pressure cycles at 10 kPa (Fig. S9). Such low fatigue behavior is attributed to the robust mechanics and strong adhesion of the electrodes and ionic film on the flexible substrate; additionally, the excellent mechanical–electrical properties of the ionic film important roles.

Figure S17 and Table S1 show performance comparisons of our sensor with other reports on traditional capacitive sensors and iontronic pressure sensors. Traditional capacitive sensors typically exhibit sensitivities and sensing ranges below 1 kPa^−1^ in the sensing range of 300 kPa, while other reports achieve a moderate balance between sensitivity and sensing range^6,17,19,28,34–51^. Our report achieves not only high sensitivity but also a wide sensing range. In addition, our sensors are prepared by screen printing, which can be further extended to large-scale commercial preparation, thus ensuring uniformity in the large-scale fabrication of sensor arrays.

### Sensor application

The sensor with high sensitivity and broad sensing range demonstrates wide practical applicability. For example, the sensor can accurately monitor the plantar pressure. Figure S10 shows that the sensor mounted inside the shoes exhibits a stable signal during the standing–sitting process. The capacitance of the sensor is much lower in the sitting state than in the standing state due to the pressure change. In addition, from the standing to sitting state, an apparently rapid increasing signal is observed due to the quick acceleration, further demonstrating the fast response time of the sensor. Figure S10b, c further shows that the sensor can accurately monitor walking status and provide a stable signal, even while running.

**Fig. 4:**
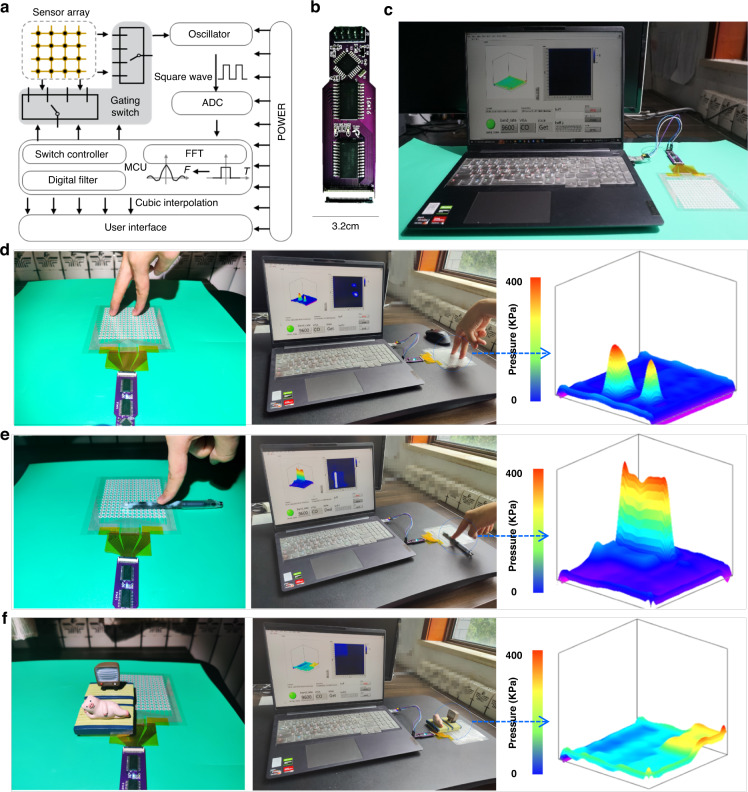
Sensor system building and spatial pressure mapping. **a** Schematic illustration and **b** digital optical image of the circuit. **c** Sensing system. **d**–**f** 2D and 3D maps of various pressures

In addition, to further enhance the practicality of the sensor, we design the sensor circuit for signal acquisition and the software for the host computer. To design the signal acquisition circuit, we connect a capacitor to an external oscillation circuit and measure the oscillation frequency changes within the circuit. When the capacitance value of the capacitor changes, the frequency of the oscillation circuit changes correspondingly. By measuring the frequency changes, the capacitance value can be calculated. However, to detect the capacitance array, it is necessary to measure each individual capacitance sensor unit. Designing a collection module for each sensor unit results in a significant waste of time and space. Therefore, we develop an array scanning circuit to scan each sensor unit. Then, the data are combined into a two-dimensional array by the MCU and packaged for display on a host computer. We utilize LabVIEW for host computer development and apply two-dimensional cubic spline interpolation to the 15 × 15 sensor array to obtain a 1500 × 1500 array. Finally, using the built-in 3D graphics control of LabVIEW, we can generate the corresponding pressure mapping. Figure 4a, b show the schematic and digital optical images of the sensor system, respectively. The pressure-induced signal is sent to the host computer through the sensor array unit selection circuit, the signal acquisition circuit and the microcontroller; thus, the 2D and 3D pressure mapping can be clearly captured (Fig. 4c). Figure 4d–f show the 3D pressure mapping of the sensing array under finger pressure, a pen located with pressure, and area pressure action, respectively. The sensing array can differentiate pressure intensity and position simultaneously, demonstrating the practical applicability of our sensor system.

**Fig. 5:**
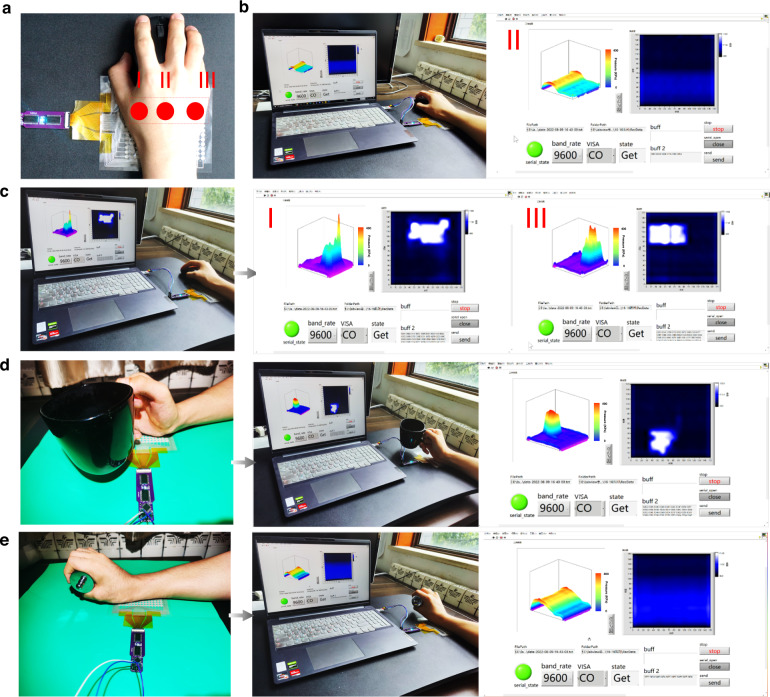
Wrist pressure of the sensor system. **a** Skeletal structure of the human palm. **b**, **c** Pressure monitoring of the sensor arrays at various states. **d**, **e** Evidence that the sensor arrays work well at an increased weight

It is necessary to monitor wrist pressure in real time since our wrist is prone to tendon sheath cysts under prolonged working conditions. Typically, the bones of the human wrist attached to the palm are divided into three parts: the navicular bone (I), the moon-shaped bone (II) and the bean-shaped bone (III) (Fig. 5a). Figure 5b, c show the 3D pressure map of the palm subjected to forces in different directions, from which the distribution of the forces can be clearly seen. When the force on the wrist is balanced, there is almost a straight line on the pressure diagram. However, when the pressure is shifted to the left (Fig. 5c, I) or right (Fig. 5c, II), a relatively sharp pressure peak can be clearly seen, proving the high sensitivity of our sensor. In particular, when we hold a cup of water (Fig. 5d) or a weight (Fig. 5e) in our hands instead of a mouse, the pressure sensor can still display the distribution and magnitude of pressure, demonstrating its ability to detect a wide range. In conclusion, with its excellent sensitivity and detection range, this iontronic flexible pressure sensing array is capable of 2D and 3D pressure detection and is expected to play an important role in human health monitoring and other applications.

## Conclusions

In summary, we have fabricated an *h*-BN-doped iontronic pressure sensor with high sensitivity and a broad sensing range through screen printing. *h*-BN is imperative for successfully printing high-performance iontronic pressure devices. On the one hand, *h*-BN is necessary for the fabrication of printable ionic ink that meets the printing requirements for large-scale manufacturing. On the other hand, the strong ion intercalation properties of *h-*BN lead to the ion pumping effect, which results in a highly sensitive sensor with various characteristics. Benefiting from such a unique structure and EDL effect, the sensor achieves a sensitivity of *S*_min_ > 261.4 kPa^−1^ over 450 kPa. More importantly, the whole sensor system is rationally integrated to achieve human health monitoring. Health monitoring, such as walking status and wrist pressure, is carefully demonstrated by the sensor system. Additionally, we believe that other 2D materials can possibly be employed in the large-scale preparation of iontronic pressure sensors and other sensors by screen printing.

## Experimental section

### Preparation of TPU-*h*-BN ionic film

During the preparation of the ion layer, 2 g of TPU powder was added to 2.5 mL of DMF. In addition, 1.0 g, 1.5 g, and 2.0 g of *h*-BN powder were separately added to 2.5 mL of DMF. The mixture was magnetically stirred at 120 °C for 60 min until TPU and *h*-BN were completely dissolved. The *h*-BN powder used in the experiment was a chemically stable pure white powder with a molecular weight of 24.81 and a particle size of 0.5 µm. The ionic liquid used was an imidazole-based ionic liquid: 1-ethyl-3-methylimidazolium bis(trifluoromethylsulfonyl)imide. The ionic liquid was a colorless and transparent liquid from the manufacturer Aladdin, with the molecular formula C_8_H_11_F_6_N_3_O_4_S_2_, a molecular weight of 391.31, a density of 1.53 g/cm^3^, and a purity of 97%. Imidazole cations and bis(trifluoromethylsulfonyl)imide anions were used as the freely mobile anions in the sensitive layer. Subsequently, 1 mL of the ionic liquid was added to the TPU solution, and the mixture was magnetically stirred at 150 °C for 30 min as the solution remained colorless and transparent. Finally, solutions containing 1.0 g, 1.5 g, and 2.0 g of *h*-BN powder were added separately, and the mixture was magnetically stirred at 150 °C for 30 min, resulting in a pure white color. After screen printing, the sensitive layer was completely solidified by heating at 80 °C for 8–10 min.

### Preparation of the iontronic pressure sensor

The electrode material was conductive silver paste. The printed silver electrode was cured by heating at 80 °C for 5 min by using screen printing on a flexible PET substrate. The average thickness of the printed silver electrode was 15 µm, and the average thickness of the ionic film was 15 µm. The sensor array was finally encapsulated by polyester double-sided tape.

### Structural, mechanical, and electromechanical characterization

Field emission scanning electron microscopy (FESEM; Quanta 450, 20 kV) was used to characterize the structural and morphological information. Mechanical properties were determined on a mechanical test machine (Zhi Qu, 990B). For each test, 5 samples were repeatedly evaluated, and the final results were obtained by averaging. For electromechanical characterization, the variable resistance, current and capacitance were recorded during the process by an electrochemical workstation (CHI 760E) and LCR impedance analyzer (IM3536 LCR meter).

## Supplementary information

Supporting information
