# Supplementary material for: Boron nitride-enabled printing of a highly sensitive and flexible iontronic pressure sensing system for spatial mapping
Source: Microsyst Nanoeng. 2023 May 26;9:68. doi: 10.1038/s41378-023-00543-x (PMC10220000; doi:10.1038/s41378-023-00543-x)
Supplement: Supplementary file 1 — Supporting information [file 41378_2023_543_MOESM1_ESM.docx]

**Boron Nitride-enabled Printing of Highly Sensitive and Flexible Iontronic Pressure Sensing System toward Spatial Mapping**

Zekun Yang^1†^, Qikai Duan^2†^, Junbin Zang^1†^, Yunlong Zhao^3^, Weihao Zheng^4^, Ran Xiao^5^, Zhidong Zhang^1^, Liangwei Hu^3^, Guirong Wu^3^, Xueli Nan^2*^, Zengxing Zhang3*, Chenyang Xue^1,3*^ and Libo Gao^3*^

^1^Key Laboratory of Instrumentation Science and Dynamic Measurement Ministry of Education, North University of China, Taiyuan 030051, China

^2^School of Automation and Software Engineering, Shanxi University, Taiyuan 030006, China

^3^Department of Mechanical and Electrical Engineering, Xiamen University, Xiamen, 361102, China

^4^School of Mechano-Electronic Engineering, Xidian University, Xi’an 710071, China

^5^Deparment of Mechanical Engineering, City University of Hong Kong, Hong Kong SAR., Kowloon 999077, Hong Kong

*****Author to whom correspondence should be addressed:

E-mail: nanxueli@sxu.edu.cn;[zhangzengxing@xmu.edu.cn;xuechenyang@nuc.edu.cn](mailto:zhangzengxing@xmu.edu.cn;xuechenyang@nuc.edu.cn);

lbgao@xmu.edu.cn;

^†^These authors contributed equally to this work.

**
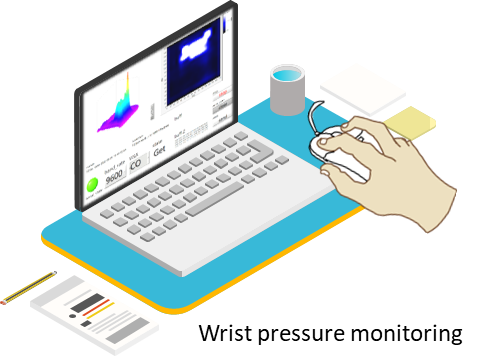
**

**Figure S1.** Schematic illustration of the wrist pressure monitoring.

**
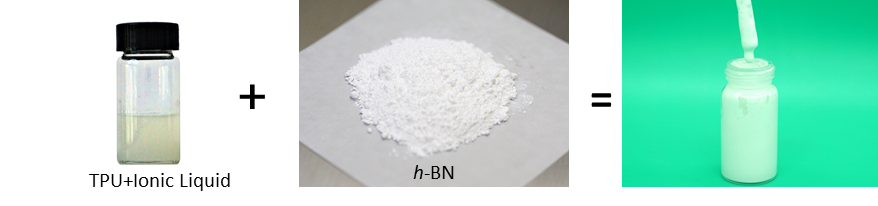
**

**Figure S2.** Composition of the thermoplastic polyether urethanes (TPU)/BN ink.

**
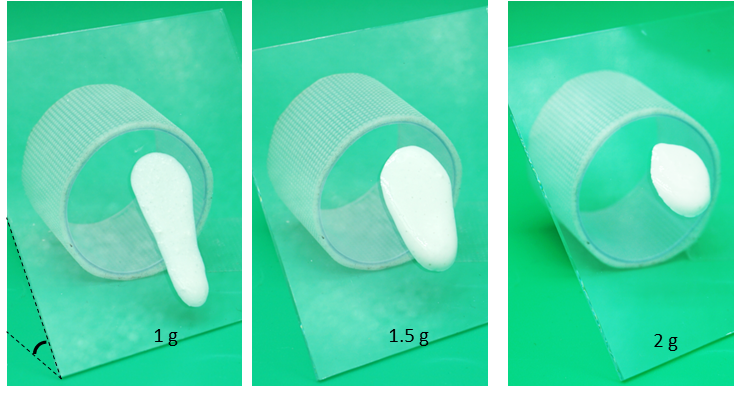
**

**Figure S3.** Various doping mass of the BN into the ionic ink.

**
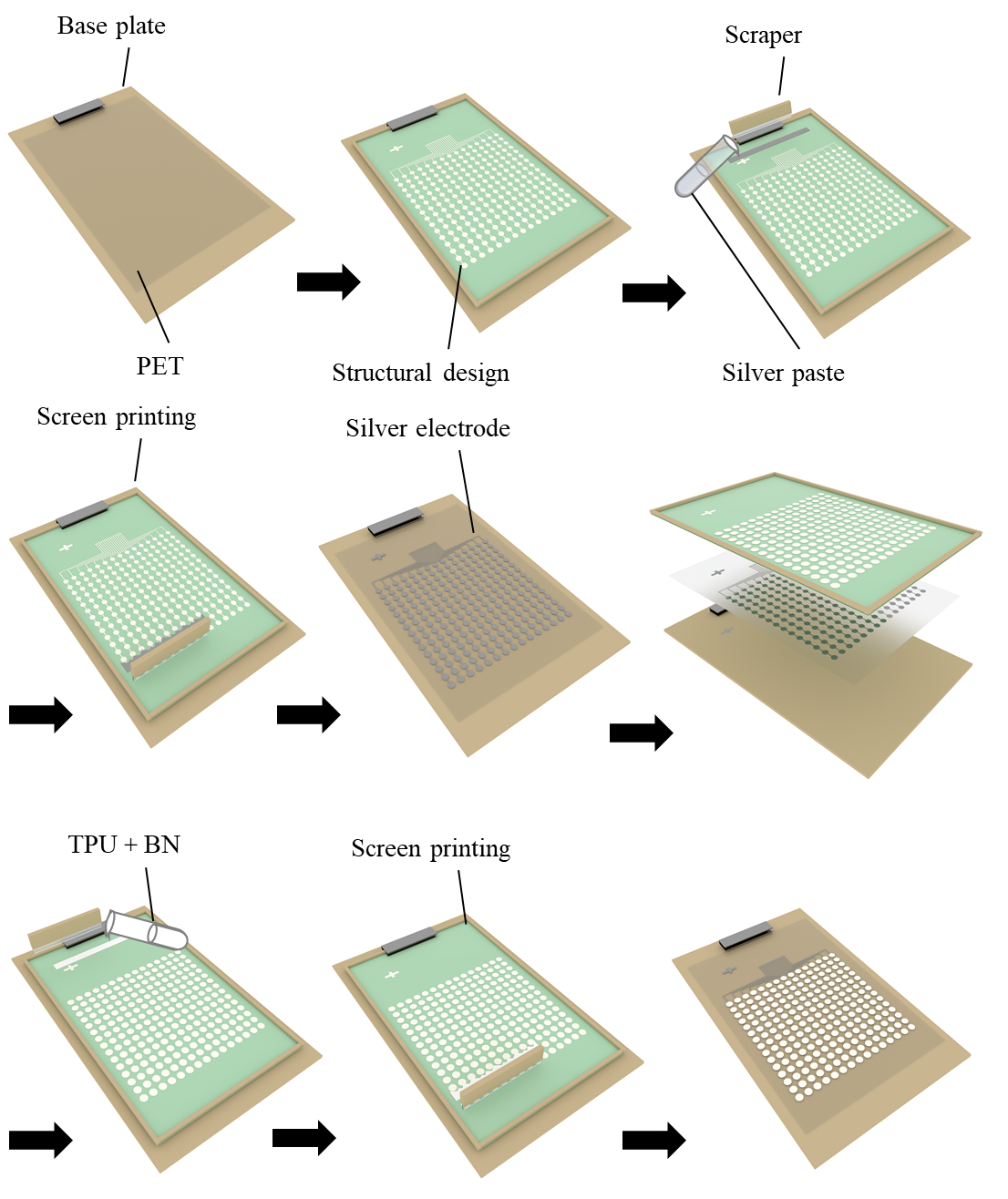
**

**Figure S4.** Schematic illustration of the fabrication of the Ag-electrode and ionic film.

**
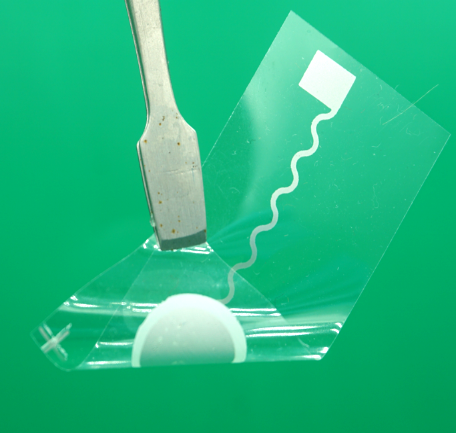
**

**Figure S5** Digital optical image of the Ag/ionic film on polyethylene terephthalate (PET) substrate

**
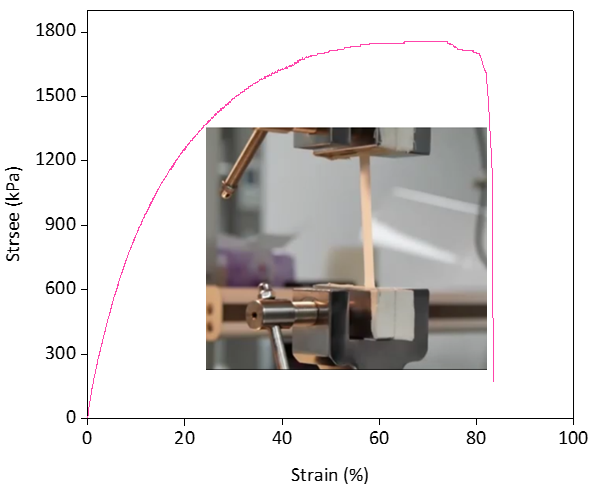
**

**Figure S6.** Mechanical tensile test of the ionic film.

**
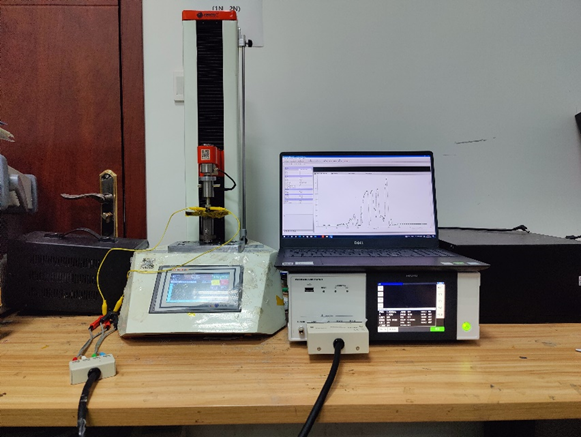
**

**Figure S7.** Configuration for the sensor characterization

**
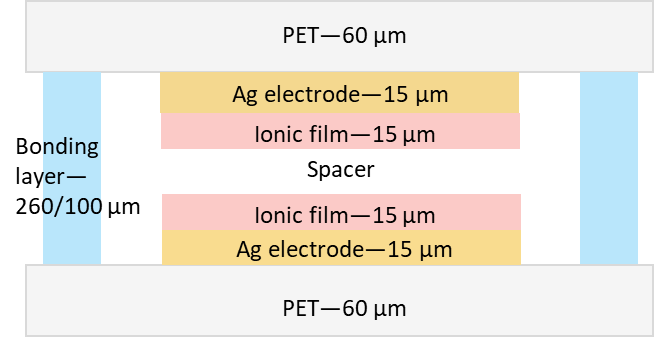
**

**Figure S8.** Device configuration of the sensor.

**
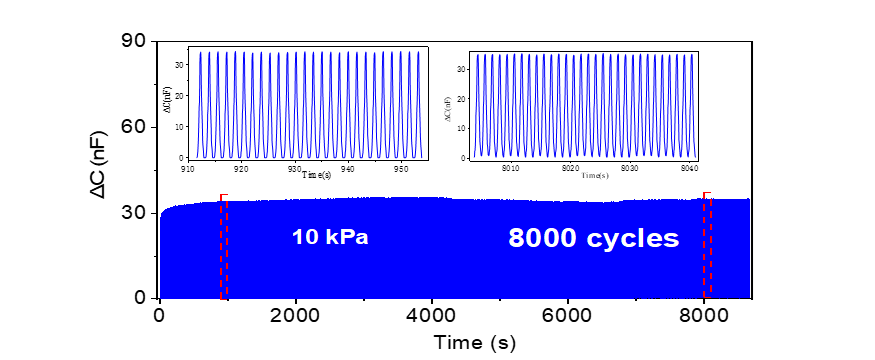
**

**Figure S9.** Long-term cycling test of the sensor at 10 kPa for 8000 cycles.

**
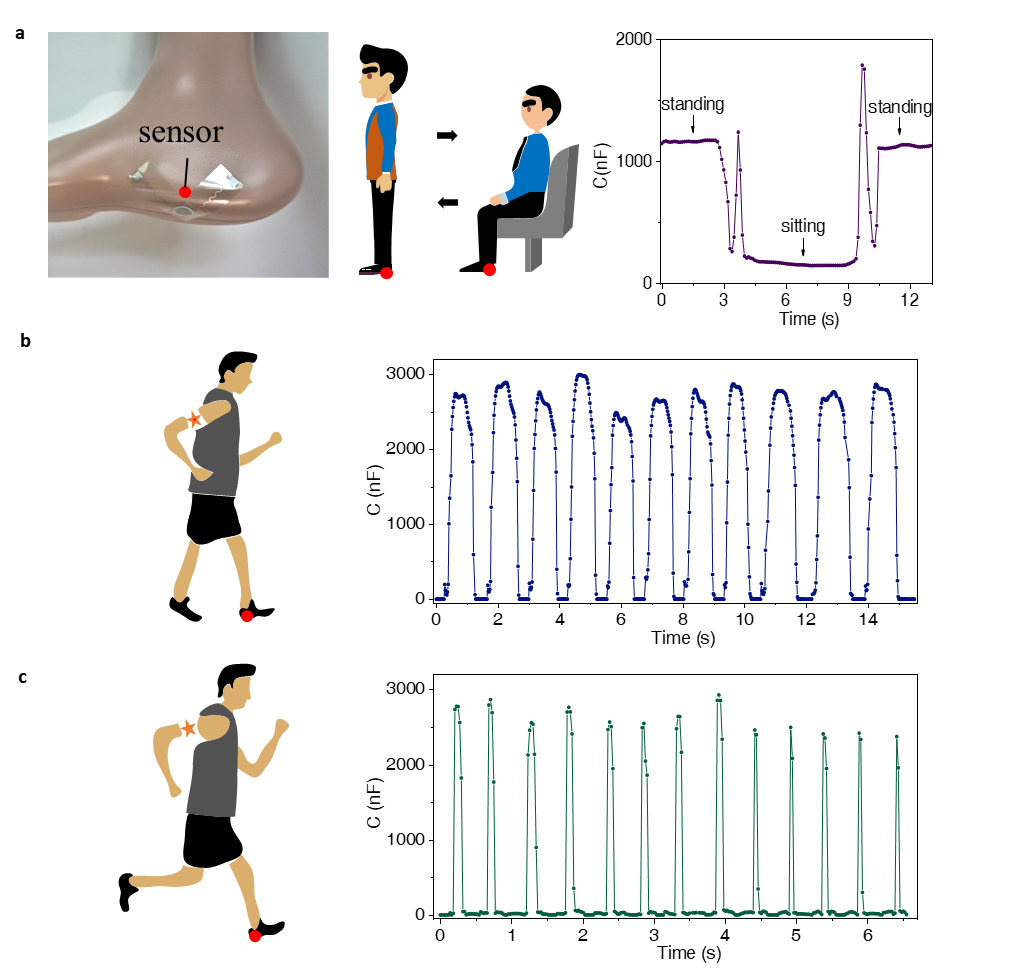
**

**Figure S10. Application of the sensor for planta pressure monitoring. a** Planta pressure of the human on sitting state. **b-c** Planta pressure of human on walking and running state.

**
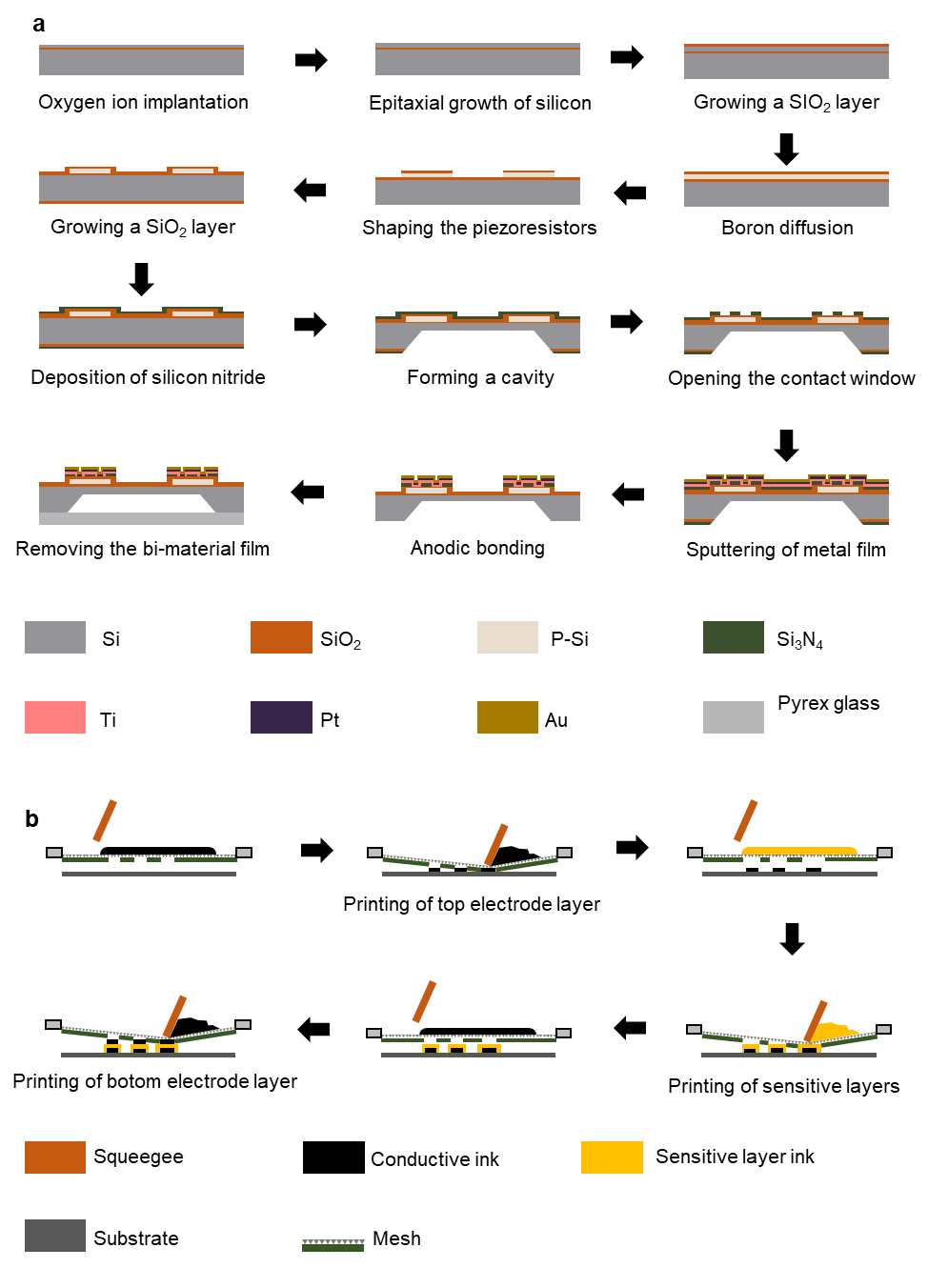
****Figure S11. Comparison of screen printing process and microelectr-mechanical systems process. a** Fabrication steps of micro pressure sensor^48^. **b** The fabrication procedure of pressure sensors utilizing screen printing technology.

**
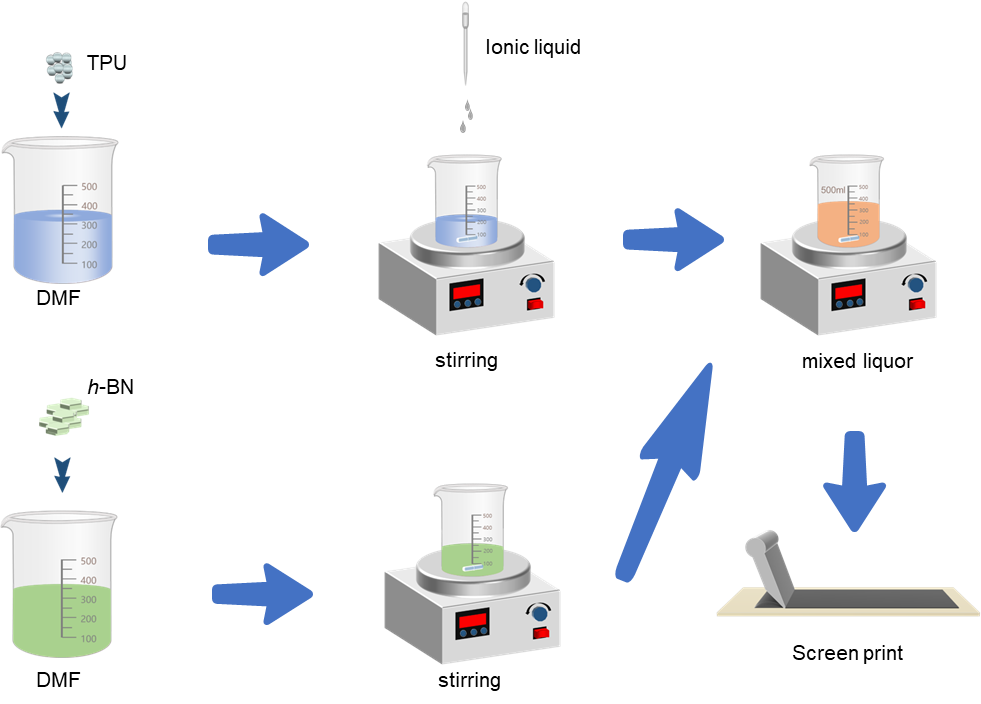
**

**Figure S12.** The preparatory procedure involved in the production of ion-sensitive materials.


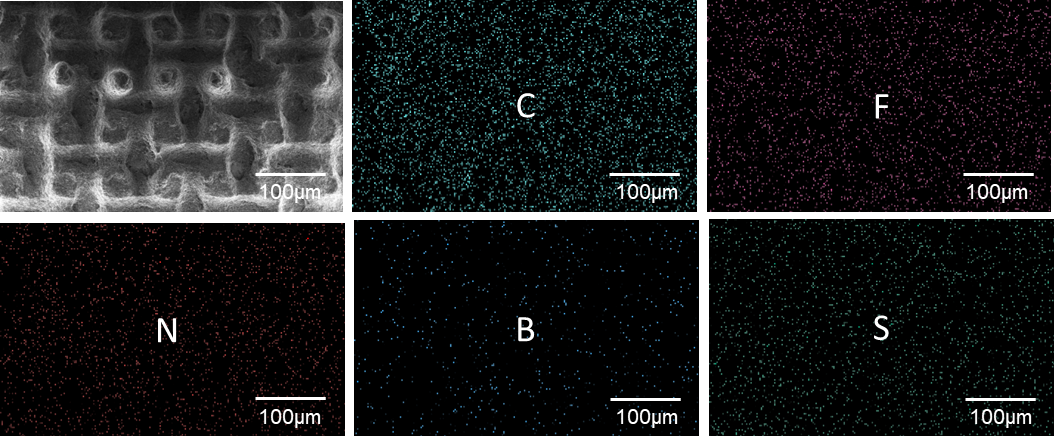
**Figure S13.** The EDS mapping images of the ionic film demonstrate a homogenous distribution of C, F, N, B, and S throughout the film, which are representative elements of TPU, h-BN, and the ionic liquid used in the film fabrication process.


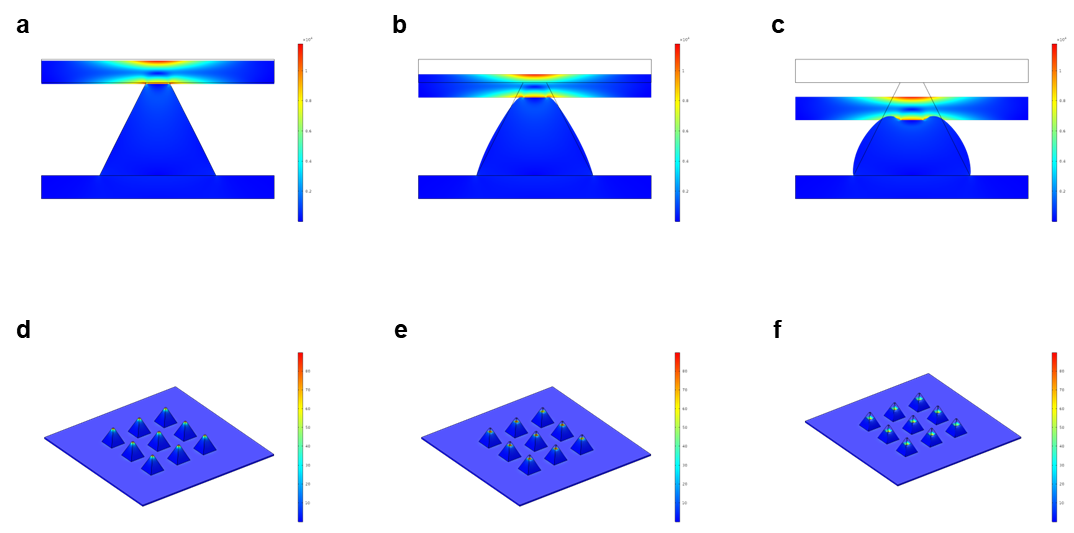
**Figure S14**. COMSOL simulations of the stress distributions on the sensor surface obtained under different pressure conditions^50^.

**Figure S15. Slump test a-c** A slump test is a method used to determine the consistency of concrete. **d** The prepared solution of the ion-sensitive layer is currently undergoing a non-flow test on a 45-degree slope. **e-g** SEM images of the front and side views of the ion-sensitive layer prepared by screen printing technique.


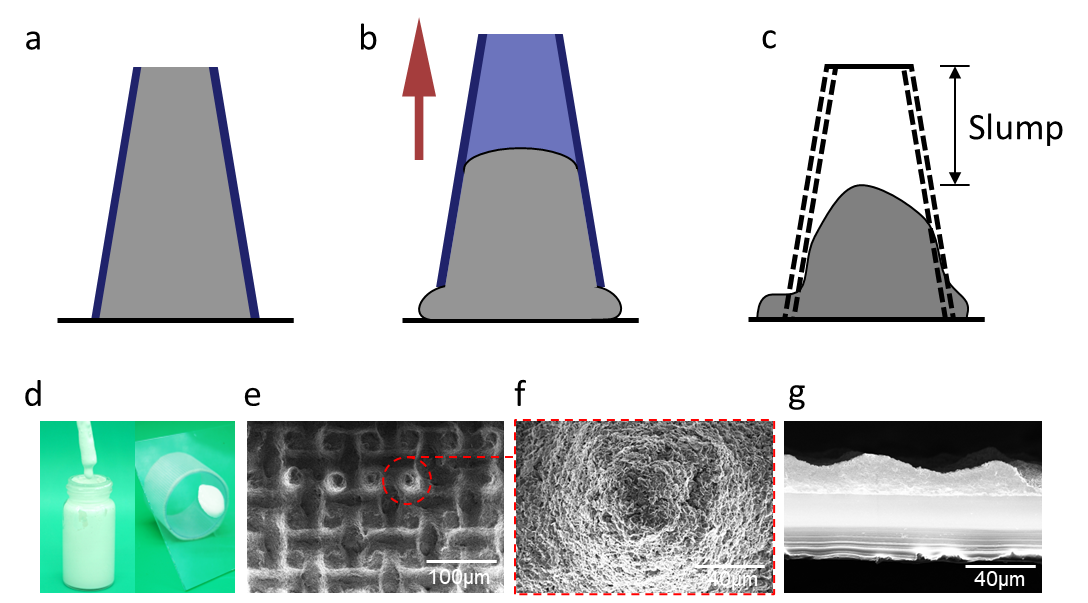

**Figure S16.** Multiple characterizations of the low detection limit.

**Figure S17**. Sensor performance Comparison.

**
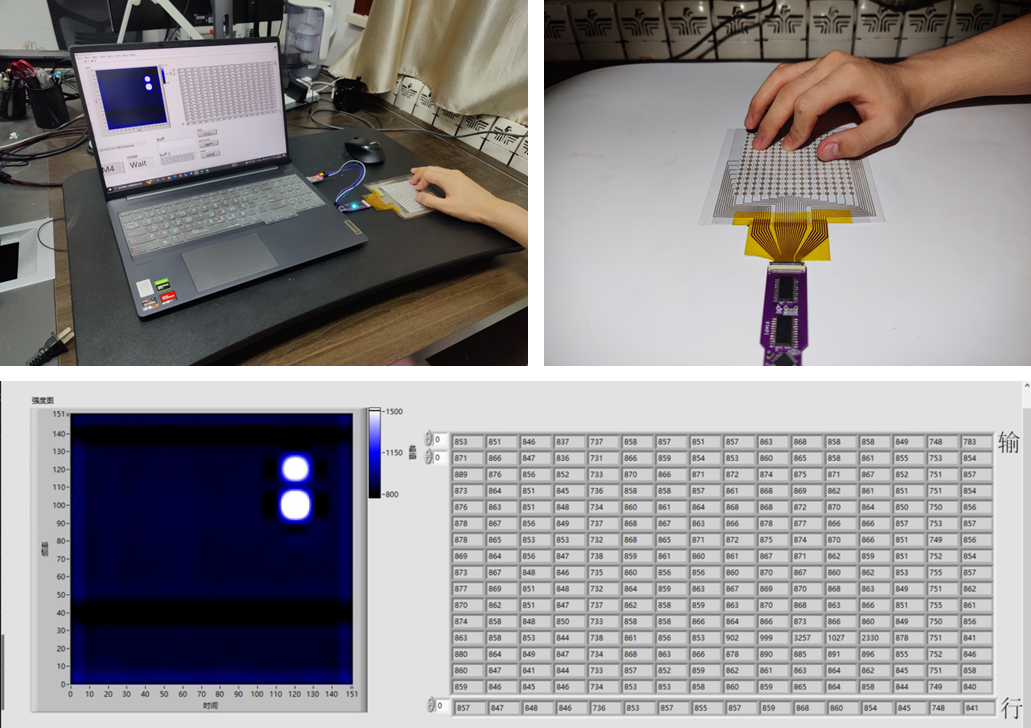
**

**Figure S18.** Static conformance testing and functional testing.

**
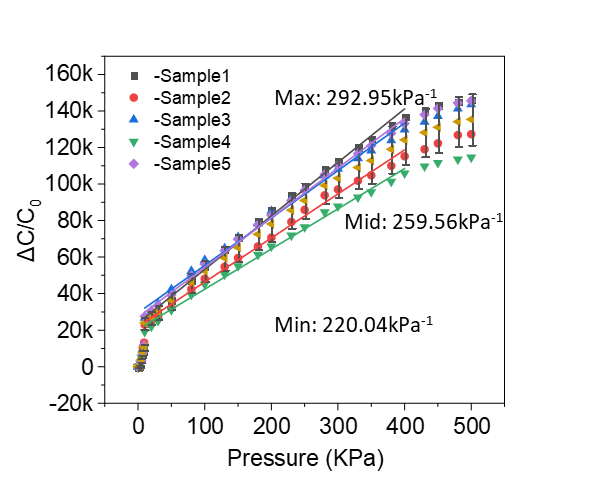
**

**Figure S19.** Performance deviation between different sensors of the array.

**
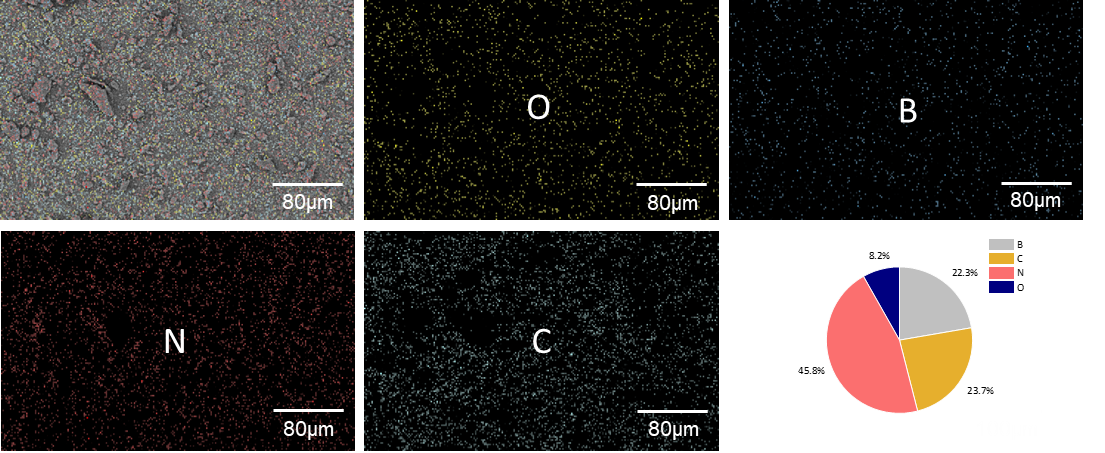
**

**Figure S20.** Characterization of field emission scanning electron microscopy morphology and energy dispersive spectroscopy analysis of h-BN powder.

**
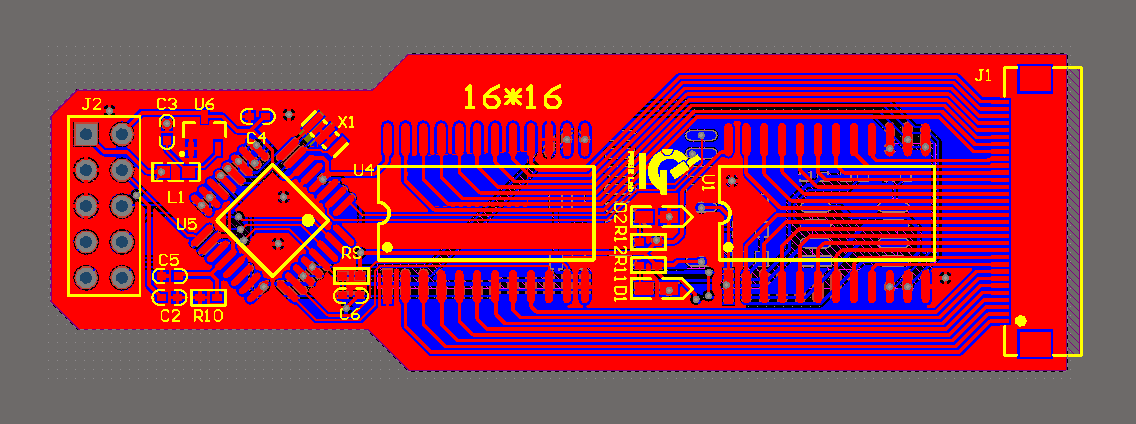
**

**Figure 21.** The acquisition circuit for a flexible capacitive array pressure sensor.

**Figure S2****2** Electrical properties testing of thin films.

**Table S1**. Performance Comparison between Traditional Capacitive Pressure Sensors and Piezoelectric Pressure Sensors.

| Ref. | TYPE | Max Sensitivity  (kPa^-1^) | Min Sensitivity  (kPa^-1^) | Pressure Range (kPa) |
| --- | --- | --- | --- | --- |
| This work | Iontronic pressure sensor | 1307.7 | 261.4 | 450 |
| Luo et al.Ref.17 | Iontronic pressure sensor | 36000 | 1237 | 50 |
| Bai et al.Ref. 6 | Iontronic pressure sensor | 49.4 | 49.4 | 500 |
| Liu et al.Ref.28 | Iontronic pressure sensor | 9280 | 628.7 | 114 |
| Chhetry et al.Ref.32 | Iontronic pressure sensor | 31.5 | 11.73 | 27.7 |
| Qiu et al.Ref.43 | Iontronic pressure sensor | 54.31 | 1.03 | 115 |
| Provust et al.Ref.45 | Iontronic pressure sensor | 35.1 | 3.5 | 5 |
| Liu et al.Ref. 41 | Iontronic pressure sensor | 547.9 | 101.8 | 110 |
| Li et al.Ref.42 | Traditional capacitive sensor | 0.815 | 0.0047 | 350 |
| Guo et al.Ref.46 | Traditional capacitive sensor | 2.9 | 1.87 | 0.85 |
| Niu et al.Ref. 40 | Traditional capacitive sensor | 6.583 | 0.125 | 1 |
| Tay et al.Ref.44 | Traditional capacitive sensor | 0.854 | 0.29 | 2.1 |
| Qiu et al.Ref. 47 | Traditional capacitive sensor | 0.063 | 0.033 | 5 |
| Xiong et al.Ref.39 | Traditional capacitive sensor | 30.2 | 0.47 | 10 |
